# Retrospective Analysis of Omicron in Minas Gerais, Brazil: Emergence, Dissemination, and Diversification

**DOI:** 10.3390/microorganisms12091745

**Published:** 2024-08-23

**Authors:** Paula Luize Camargos Fonseca, Isabela Braga-Paz, Luiza Campos Guerra de Araújo e Santos, Rillery Calixto Dias, Carolina Senra Alves de Souza, Nara Oliveira Carvalho, Daniel Costa Queiroz, Hugo José Alves, João Locke Ferreira de Araújo, Filipe Romero Rebello Moreira, Mariane Talon Menezes, Diego Menezes, Aryel Beatriz Paz e Silva, Jorge Gomes Goulart Ferreira, Talita Emile Ribeiro Adelino, André Felipe Leal Bernardes, Natália Virtude Carobin, Renée Silva Carvalho, Carolina Zaniboni Ferrari, Natália Rocha Guimarães, Ludmila Oliveira Lamounier, Fernanda Gil Souza, Luisa Aimeé Vargas, Marisa de Oliveira Ribeiro, Monica Barcellos Arruda, Patricia Alvarez, Rennan Garcias Moreira, Eneida Santos de Oliveira, Adriano de Paula Sabino, Jaqueline Silva de Oliveira, José Nélio Januário, Felipe Campos de Melo Iani, Renan Pedra de Souza, Renato Santana Aguiar

**Affiliations:** 1 Laboratório de Biologia Integrativa, Departamento de Genética, Ecologia e Evolução, Instituto de Ciências Biológicas, Universidade Federal de Minas Gerais, Belo Horizonte 31270-901, Brazil; camargos.paulaluize@gmail.com (P.L.C.F.); isabela-lorraine2010@hotmail.com (I.B.-P.); luizacamposgs@gmail.com (L.C.G.d.A.e.S.); rillery_calixto@hotmail.com (R.C.D.); dcqbioufmg@gmail.com (D.C.Q.); alves.hugo.j@gmail.com (H.J.A.); joaolocke.bio@gmail.com (J.L.F.d.A.); t.menezes.diego@gmail.com (D.M.); arypaz06@gmail.com (A.B.P.e.S.); ferreira_jgg@yahoo.com (J.G.G.F.); nandags10@hotmail.com (F.G.S.); rennangm@gmail.com (R.G.M.); 2 Subsecretaria de Vigilância em Saúde, Secretaria de Estado de Saúde de Minas Gerais, Belo Horizonte 31585-200, Brazil; carol.senraas@gmail.com (C.S.A.d.S.); reneecarv@gmail.com (R.S.C.); carolinazferrari@hotmail.com (C.Z.F.); luisaaimee@outlook.com (L.A.V.); jaquelinebmedica@hotmail.com (J.S.d.O.); 3 Núcleo de Ações e Pesquisa em Apoio Diagnóstico-Nupad, Faculdade de Medicina, Universidade Federal de Minas Gerais, Belo Horizonte 30130-100, Brazil; nara@nupad.medicina.ufmg.br (N.O.C.); nelio@nupad.medicina.ufmg.br (J.N.J.); 4 Departamento de Genetica, Instituto de Biologia, Universidade Federal do Rio de Janeiro, Rio de Janeiro 21941-902, Brazil; filiperomero2@gmail.com (F.R.R.M.); marianetalon@gmail.com (M.T.M.); 5 Fundacao Ezequiel Dias (FUNED), Belo Horizonte 30510-010, Brazil; talitaemile@yahoo.com.br (T.E.R.A.); andrefelipeleal@yahoo.com.br (A.F.L.B.); natyroguiman@yahoo.com.br (N.R.G.); ludmila.lamounier@funed.mg.gov.br (L.O.L.); felipeemrede@gmail.com (F.C.d.M.I.); 6 Laboratório Institucional de Pesquisa em Biomarcadores, Laboratório de Hematologia Clínica, Departamento de Análises Clínicas e Toxicológicas; Faculdade de Farmácia, Universidade Federal de Minas Gerais, Belo Horizonte 31270-901, Brazil; natyvirca@gmail.com (N.V.C.); adriansabin01@gmail.com (A.d.P.S.); 7 Institute of Technology in Immunobiology Bio-Manguinhos, Oswaldo Cruz Foundation/Fiocruz, Rio de Janeiro 21040-900, Brazil; mribeiro@bio.fiocruz.br (M.d.O.R.); monica.arruda@bio.fiocruz.br (M.B.A.); palvarez@bio.fiocruz.br (P.A.); 8 Secretaria Municipal de Saúde de Belo Horizonte, Belo Horizonte 30130-012, Brazil; eneida.oliveira@pbh.gov.br; 9 Instituto D’OR de Pesquisa e Ensino, Rio de Janeiro 22281-100, Brazil

**Keywords:** VOC Omicron, SARS-CoV-2, subvariants, epidemiological scenario, genomic surveillance

## Abstract

Brazil is one of the countries most affected by COVID-19, with the highest number of deaths recorded. Brazilian Health Institutions have reported four main peaks of positive COVID-19 cases. The last two waves were characterized by the emergence of the VOC Omicron and its sublineages. This study aimed to conduct a retrospective surveillance study illustrating the emergence, dissemination, and diversification of the VOC Omicron in 15 regional health units (RHUs) in MG, the second most populous state in Brazil, by combining epidemiological and genomic data. A total of 5643 confirmed positive COVID-19 samples were genotyped using the panels TaqMan SARS-CoV-2 Mutation and 4Plex SC2/VOC Bio-Manguinhos to define mutations classifying the BA.1, BA.2, BA.4, and BA.5 sublineages. While sublineages BA.1 and BA.2 were more prevalent during the third wave, BA.4 and BA.5 dominated the fourth wave in the state. Epidemiological and viral genome data suggest that age and vaccination with booster doses were the main factors related to clinical outcomes, reducing the number of deaths, irrespective of the Omicron sublineages. Complete genome sequencing of 253 positive samples confirmed the circulation of the BA.1, BA.2, BA.4, and BA.5 subvariants, and phylogenomic analysis demonstrated that the VOC Omicron was introduced through multiple international events, followed by transmission within the state of MG. In addition to the four subvariants, other lineages have been identified at low frequency, including BQ.1.1 and XAG. This integrative study reinforces that the evolution of Omicron sublineages was the most significant factor driving the highest peaks of positive COVID-19 cases without an increase in more severe cases, prevented by vaccination boosters.

## 1. Introduction

In Brazil, from January 2020 to June 2023, there were nearly 38 million confirmed cases and 700,000 deaths [1]. The epidemic’s progression in Brazil has been marked by four significant waves, each characterized by a continuous rise in case numbers, leading to new epidemiological situations [1]. These waves or peaks of infections are linked to the emergence of new variants that provide the virus with certain advantages, such as increased transmissibility, virulence, or an improved ability to evade the immune system [2,3,4].

The initial wave peaked in July 2020, resulting from the virus’s introduction in Brazil and the circulation of lineages B.1.1.28 and B.1.1.33 [5,6]. The second wave, reaching its peak in March 2021, had significant repercussions, particularly in Amazonas state, attributed to the emergence of the Gamma variant, designated a “variant of concern” (VOC) [7]. In April 2021, the Delta variant surfaced in the country, eventually supplanting the Gamma variant and becoming the predominant strain nationally by November 2021 [8,9]. Notably, despite the introduction of the Delta variant amidst high immunization rates (both natural and vaccine-induced), it did not lead to a substantial surge in new cases [9]. Consequently, by the beginning of 2021, Brazil reported the lowest number of new cases and deaths up to that point, which contributed to the relaxation of preventive measures, creating a conducive environment for the rapid spread of the Omicron VOC upon its introduction in December [10].

The third wave peaked in January 2022 following the introduction of Omicron BA.1 [11,12]. Since this subvariant emerged, the virus has undergone further evolution, resulting in numerous descendant and recombinant lineages associated with the increasing number of COVID-19 cases. The epidemiological situation experienced further changes from March 2022 onward due to the circulation of the BA.2 sublineage. Despite the continued circulation of new lineages, the number of cases and deaths decreased due to the increase in vaccination rates in Brazilian cities [12,13]. After this period, two new waves altered the epidemiological landscape once more, driven by the circulation of new sublineages of the Omicron variant. The fourth wave, starting in June 2022, was caused by the circulation of the BA.4 and BA.5 sublineages [14]. The fifth wave commenced in November 2022 with the introduction of recombinant lineages BQ.1 and BE.9 [14], followed by the circulation of the XBB variant, a recombinant of the Omicron variant, giving rise to other subvariants. Currently, the variant of interest (VOI) JN.1 and its sublineages, the recombinant XDR, and the VOI XBB.1.5 prevail in the Brazilian epidemiological scenario [15].

Genomic monitoring of circulating viruses becomes essential to investigate the variants’ diversity, distribution, and evolutionary patterns, estimate transmission rates, and assist in tracking outbreaks. This information, when associated with epidemiological data, can provide strategic tools for public health authorities and influence decisions towards combating COVID-19. In addition, genomic information is remarkably important during periods of intense viral circulation and rapid increase in the number of cases, which are conducive to the emergence and selection of new mutations and possibly new variants associated with more severe cases and vaccine escape [10,12].

In Minas Gerais (MG) state, grappling with the spread of SARS-CoV-2 and the circulation of VOCs have posed a significant challenge for public health policies addressing the COVID-19 pandemic. As one of the country’s most populous and expansive states, MG has faced unique difficulties. Its extensive population, vast territory, and numerous international passenger destinations throughout the pandemic have made it a complex arena for managing the impact of COVID-19. Additionally, the state ranked as the third most affected during the COVID-19 pandemic [5,16]. Furthermore, MG is the Brazilian state with the highest number of municipalities (853 counties), making it an excellent model for evaluating the spread of the COVID-19 epidemic to inland areas. Herein, we present a retrospective study showing the dissemination and diversification of VOC Omicron in MG, combined with epidemiological and genomic data.

A total of 5643 samples from 15 regional health units (RHUs) (covering almost 54% of the state) were genotyped during the Omicron waves. An increase in the number of cases was observed near the introduction date of new Omicron lineages. However, despite the rise in cases, the number of deaths did not increase, which may be attributed to vaccination and booster doses in the population. A total of 253 new genomes were used to estimate the introduction date of the four subvariants in the state. Different lineages were identified, indicating the diversity of Omicron VOC lineages circulating in the state. The results found in this study enable public health entities to monitor the spread of COVID-19 in the state and facilitate decision making to contain its advancement.

## 2. Materials and Methods

### 2.1. Genomic Surveillance Observatory and Study Design

The *Observatório de Vigilância Genômica do Estado de* Minas Gerais (OViGen) is a real-time monitoring project of circulating SARS-CoV-2 in MG. The OViGen is a partnership among Minas Gerais State Health Secretary (SES/MG) and other public and private laboratories: the state reference laboratory *Fundação* Ezequiel Dias (FUNED), *Laboratório de Biologia Integrativa* (LBI), and The *Núcleo de Ações e Pesquisas em Apoio Diagnóstico* (NUPAD) from the *Universidade Federal de* Minas Gerais (UFMG).

**Figure 1:**
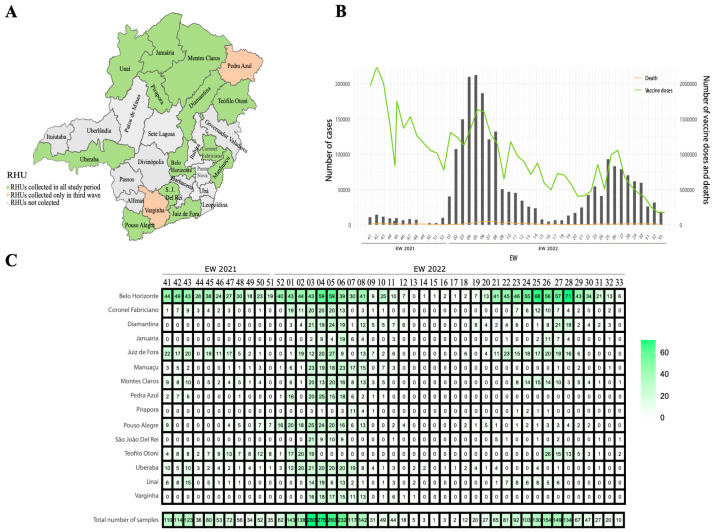
The epidemiological status of SARS-CoV-2 in the state of MG. (**A**) Regional health units (RHUs) from MG state sampled in our study. Two RHUs were sampled only in the third wave (orange color). (**B**) Number of cases, deaths, and vaccination status per epidemiological week (EW) in MG during the study period. (**C**) Samples retrieved by RHUs with SARS-CoV-2 lineages classification by on our genotyping strategy.

We conducted a longitudinal study from 10 October 2021 to 28 August 2022 using nasopharyngeal swabs with positive SARS-CoV-2 RT-qPCR samples (between the epidemiological weeks 41/2021 and 33/2022) collected in different regional health units (RHUs). Fifteen of the twenty-eight RHUs of MG state were included in our study. The RHUs Belo Horizonte, Coronel Fabriciano, Diamantina, Januária, Juiz de Fora, Manhuaçu, Montes Claros, Pedra Azul, Pirapora, Pouso Alegre, São João Del Rei, Teófilo Otoni, Uberaba, Unaí, and Varginha were selected due to their geographic location, since they can border other states or have a large population flow or transport traffic (Figure 1A).

Our study was approved by the Research Ethics Committee (CAAE: 33202820.7.1001.5348).

### 2.2. Genotyping and SARS-CoV-2 Whole-Genome Sequencing

A total of 5,643 positive samples collected between the 41st (2021) and 33rd (2022) EW were genotyped using specific primers for mutations localized in the Spike gene. At the beginning of our study, the mutations N501Y and L452R, from the TaqMan SARS-CoV-2 Mutation Panel (Thermo Fisher, Waltham, MA, USA), were used to classify samples as Delta variant (see [9]). Samples not amplified for the L452R position were also tested for specific mutations found in the Omicron variant. In that case, the genotyping was conducted using the 4Plex SC2/VOC Bio-Manguinhos (Fiocruz, Rio de Janeiro, RJ, Brazil) kit, with the primer for the Spike polymorphisms DelH69 and V70 [17], capable of distinguishing between the Omicron sublineages BA.1 (deleted) and BA.2 (nondeleted).

One sample by region and by EW was selected for whole-genome sequencing. All DNA libraries were assembled using the QIAseq SARS-CoV-2 Primer Panel—QIAGEN (Hilden, Germany), with the ARTIC primer pools: V4.0 and V4.1 [18,19], quantified by the QIAseq Library Quant Assay kit—QIAGEN and sequenced on the MiSeq (Illumina, San Diego, CA, USA) platform with v3 (600 cycles) cartridges.

All sequencing data went through a custom pipeline for processing and to generate the consensus genome as described by [20]. Low-depth bases (less than 20× sequencing depth) were not considered for further analysis as well as sequences with less than 70% genome coverage. Sequences that fulfill all requirements were classified using Pangolin v.4.1.1 [21] or Nextclade v.2.4.2 [22] tools.

### 2.3. Phylogeographic Reconstructions

In this study, we constructed five different datasets to confirm lineage classification and further contextualize the dynamics of the introduction of Omicron BA.1 (Nextclade 21K), BA.2 (Nextclade 21L), BA.4 (Nextclade 22A), and BA.5 (Nextclade 22B) clades in MG. The first dataset comprised all genomes generated in our study and all public reference genomes classified by NextStrain as Omicron clades (accessed on 2 January 2023), totaling 1750 genomes. This dataset was aligned using Minimap2 [23], and a maximum likelihood phylogeny was generated in IQ-tree v2.0.3 [24] with the GTR + F + I + G4 nucleotide substitution model and the “---polytomy” flag to collapse short branches. The tree was rooted in the oldest sequence present in the dataset.

The other datasets pertained to the dynamics of the introduction of these subvariants in MG. First, for each dataset, we included all BA.1, BA.2, BA.4, or BA.5 sequences from Nextstrain builds (accessed on 23 May 2023: BA.1 = 4968, BA.2 = 3443, BA.4 = 2617, and BA.5 = 6156). Second, we selected random genomes from each Brazilian state (26 states) proportionally to the number of estimated Omicron weekly cases during our study period [9]. The number of cases per week from each state was collected from the https://opendatasus.saude.gov.br database (accessed on 9 November 2023). Whenever sampling gaps were detected, all available data were included. The number of genomes and cases, as well as the proportion of Omicron cases each week, are available in Supplementary Table S1. The third step for phylogenetics was to construct maximum likelihood phylogenies for each subvariant dataset, as described previously [2,9]. Each tree was submitted to TempEst v1.5 [25] to identify genomes with inconsistent temporal signals. Outliers with residuals deviating 1.5-fold from the interquartile range were excluded. The genomes with consistent temporal signals were used in the next steps of the analysis.

The fourth step involved using the BEAST Thorney for molecular clock phylogenetic reconstruction. The analysis was performed considering the replacement model HKY + G4, a strict molecular clock model, and the previous skygrid tree prior. For each dataset, multiple Markov chain Monte Carlo (MCMC) runs were performed with 200 million generations, sampling every 20,000 steps, removing 10% of chains as burn-in. The software Tracer v1.7.1 was used to evaluate the convergence of the tree (effective sample size > 200). Logs and trees were combined with logcombiner.

The final step was to perform phylogeographic reconstruction of each sublineage (BA.1, BA.2, BA.4, and BA.5) in MG. This was carried out using a discrete asymmetric model with nine different states: four Brazilian geographic regions (north, northeast, midwest, and south), and four states from the southeast region (Espírito Santo, Minas Gerais, Rio de Janeiro, and São Paulo) and international sequences from the Nextclade. For each dataset, independent analyses were carried out until a good convergence was achieved. Maximum clade credibility trees were inferred for all datasets with TreeAnnotator v1.10.2.

### 2.4. Epidemiological and Clinical Data Analysis

Epidemiological data were obtained from SES-MG using the databases E-SUS, *Sistema de Informações de Vigilância Epidemiológica* (SIVEP-gripe), and *Sistema de Informações do Programa Nacional de Imunizações* (SI-PNI) in compliance with patient data protection laws. Data related to sex, age, clinical outcome, vaccine strategy, and vaccination doses and dates were used in our analyses. The number of COVID-19 cases, deaths, and vaccinations were obtained from coronavirus https://coronavirus.saude.mg.gov.br/dadosabertos and https://saude.gov.br (accessed on 10 May 2023).

## 3. Results

### 3.1. Epidemiological Status of SARS-CoV-2 in MG State

We monitored the epidemiological situation of COVID-19 in 15 RHUs of MG from EW 41 (2021) to EW 33 (2022) (Figure 1A). In EW 41, the number of vaccine doses administered was the highest in the entire study period. After that week, the number of cases and deaths was the lowest in the series studied. By that time, the Delta lineage was the most prevalent described in MG. However, in the last two EWs of 2021, we observed an increase in the number of cases and deaths, which may be related to the emergence of BA.1 in MG. Similarly, another increase in cases can be correlated with the appearance of BA.4 and BA.5 in the state (Figure 1B). During our study, we aimed to retrieve as much data and as many samples as possible per RHU and EW in MG to assess the dispersion of Omicron subvariants. However, in some RHUs, it was not possible to find samples in all EWs. The RHU with the highest number of samples was Belo Horizonte, the state capital, while other RHUs, such as Januária and São João Del Rei, had few samples evaluated (Figure 1C). The absence of samples in some RHUs per EW may be due to a reduction in COVID-19 diagnoses and the increase in vaccination rates.

Moreover, there was a variation in number of samples per EW (Figure 1C), with the highest number of samples found in EW 1 to 8 of 2022. This pattern corresponds to the confirmed cases curve, with the highest number of samples being collected during the period of the highest number of cases. We also observed that these numbers of samples varied by RHU (Figure 1A), with the Belo Horizonte RHU having the highest number of samples (32.9%). The RHUs of Januária, Pirapora, São João Del Rei, Unaí, and Varginha had fewer than 100 samples analyzed.

### 3.2. Delta to Omicron Replacement and Its Sublineages in MG

**Figure 2:**
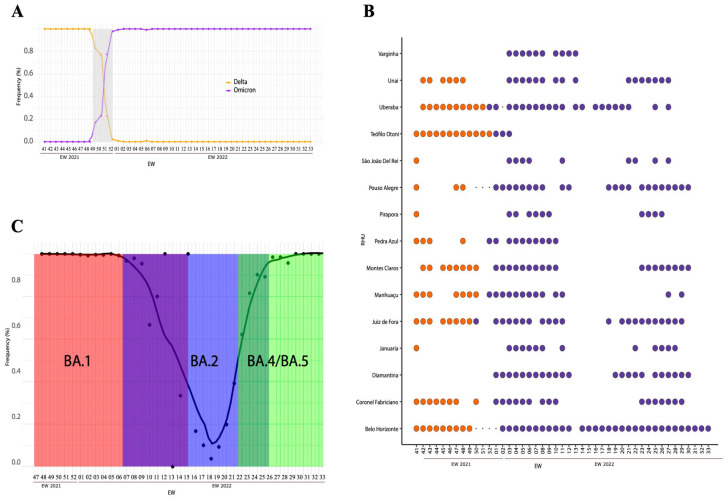
Distribution of SARS-CoV-2 lineages in MG. (**A**) Replacement of Delta to Omicron in MG state. The color orange represents Delta, while purple represents VOC Omicron. (**B**) Replacement period between Delta and VOC Omicron by RHU per EW. Blank spaces indicate the absence of samples in that RHU and EW. The orange color represents the VOC Delta, while the purple represents VOC Omicron. (**C**) Frequency of the H69/V70 deletion present in the samples investigated throughout the study. Transition periods between the predominance of BA.1 to BA.2 and from BA.2 to BA.4/BA.5 are indicated by the overlapping colors.

Our results showed a shift from Delta to Omicron in MG during the EWs 50/2021 to 01/2022 (Figure 2A). The first Omicron samples were identified in EW 50/2021 in the RHUs of Belo Horizonte, Juiz de Fora, and Pouso Alegre, suggesting that the introduction of the variant occurred during this period in the state. In the following weeks (51/2021 to 01/2022), there was a turnover between the Delta and Omicron variants. In EW 52/2021, the VOC Omicron accounted for 70% of the samples classified in the RHU Belo Horizonte, 90% in the RHU Varginha, and 100% in other RHUs with positive samples during this period (Figure 2B). In the EW 03/2022, the VOC Omicron accounted for 100% of the positive COVID-19 cases in MG (Figure 2A). The increase in Omicron cases between EWs 50/2021 and 01/2022 coincides with the change in the epidemiological scenario depicted in Figure 1B.

After Omicron became prevalent in all RHUs analyzed, new mutations and the emergence of subvariants were recorded. To identify the circulation of subvariants in the state, we performed genotyping for the presence of VOC Omicron-specific mutations. During EWs 50-08, the BA.1 subvariant was predominant throughout the state. In the first EWs of 2022, the BA.2 subvariant was also identified. However, this subvariant was detected in 100% of the samples only in EW 14 (April 2022) and continued to be detected, albeit at lower rates until EW 25, alongside other subvariants. From EW 25 onwards, an increase in the frequency (above 80%) of subvariants BA.4 and BA.5 was observed until the end of the study period. BA.1 and BA.2 circulated for more than 18 weeks in the state, making them the subvariants with the longest circulation period among Omicron lineages (Figure 2C). Information about each genotyped sample is available in Supplementary Table S2.

### 3.3. Epidemiological Scenario during the Turnover of Variants and Subvariants

We had access to the clinical outcomes of 3481 patients whose samples were genotyped and sequenced in our study. Of these, 591 were classified as VOC Delta, 351 as BA.2, and 2493 as possible BA.1, BA.4, or BA.5 (referred as “no BA.2” in Table 1). The majority of samples were from females (*n* = 2.029—59%) and classified in subvariants BA.1, BA.4, and BA.5. The majority of cases evaluated were aged between 30 and 60 years (*n* = 1.776—52%), followed by the group aged 0 to 29 years (*n* = 885—26%) concentrated in the group of subvariants BA.1, BA.4, and BA.5 (“no BA.2”). The group with the highest death rate was from subvariants BA.1, BA.4, and BA.5 (“no BA.2”) (*n* = 45—1.8%), followed by VOC Delta (*n* = 41—6.9%). A summary of epidemiological data evaluated is available in Table 1.

**Figure 3:**
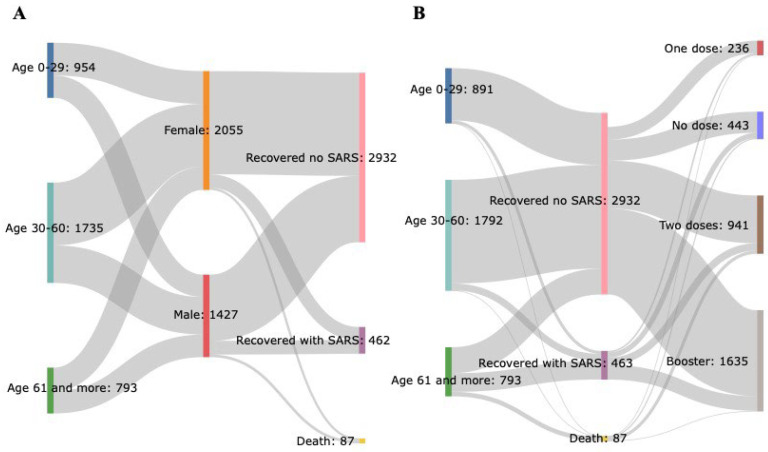
Clinical outcomes and vaccination status for confirmed Omicron cases. (**A**) Clinical outcomes classified by sex and age. (**B**) Clinical outcomes classified by age and vaccination status.

Regarding the clinical outcome, most genotyped cases recovered from COVID-19, with 84.23% recovered without any worsening, 13.27% recovering despite having severe acute respiratory syndrome (SARS), and 2.49% being fatal cases (Figure 3A). The main factor contributing to clinical outcome was age. Most cases classified in the recovered group that developed SARS were individuals aged 61 or over (*n* = 301—65%), and individuals who died (*n* = 73—83.9%). Furthermore, most individuals aged 61 or older who died had received at least two doses of vaccine (*n* = 48—55.1%) (Figure 3B). Nevertheless, the number of deaths was small compared to the number of samples evaluated (2.5%), indicating that vaccination effectively reduces the number of deaths and the development of severe clinical conditions, even in the presence of Omicron subvariants.

### 3.4. Omicron Lineages in Circulation in MG State

**Figure 4:**
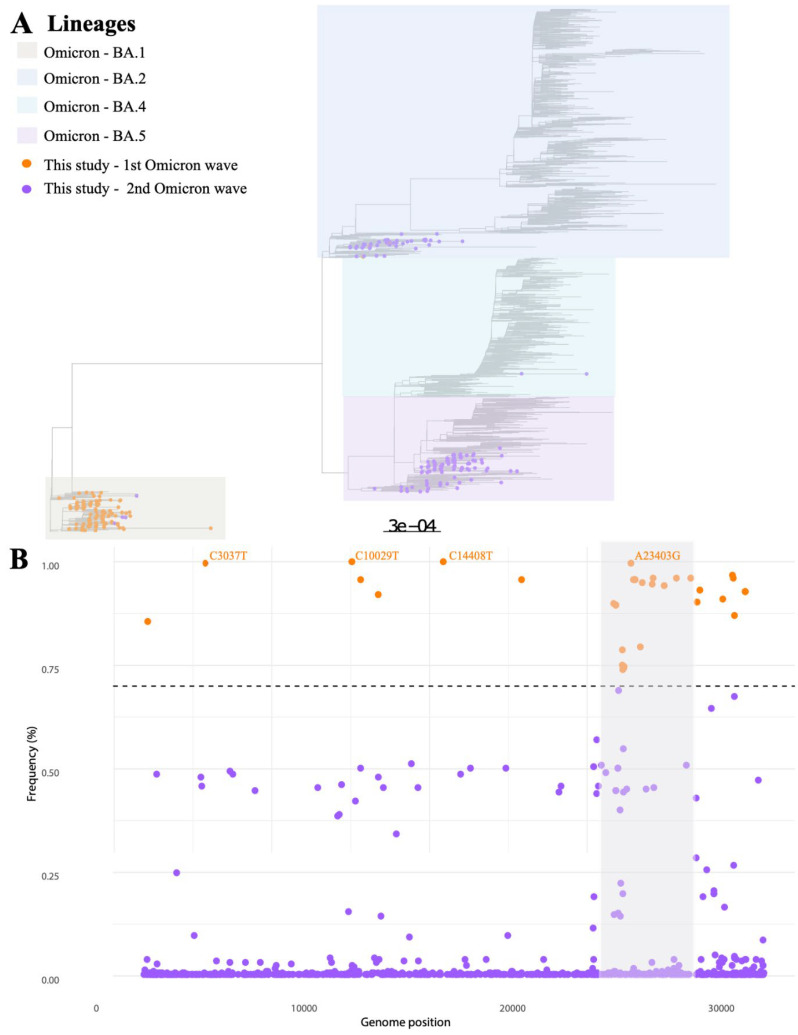
Diversity of Omicron subvariants in MG state. (**A**) Maximum likelihood phylogeny using the VOC Omicron NextStrain Global dataset as reference. The circles represent the genomes generated in our study (*n* = 253) according to the Omicron wave which they were collected. Each Omicron sublineage (BA.1, BA.2. BA.4, and BA.5) is represented by a different color. (**B**) Synonymous mutation profile of the sequenced genomes. More than 858 mutations were identified. Orange circles represent synonymous mutations found in at least 70% of the genomes, with only four presented in all genomes (100%). The mutations are labeled next to the circles. Purple circles represent mutations found at low frequency in the genomes generated. The gray shading indicates the position of the Spike gene in the SARS-CoV-2 genome.

A total of 253 new whole genomes were generated in our study. Genome coverage varied from 66.35% to 99.75% (median = 95.51%), and mean sequencing depth varied from 3.418× to 900.388× (median = 448.802×). According to Pangolin, Nextclade, and our phylogenetic analysis (Figure 4A), we identified 126 samples from 10 different lineages from 21K clade (Omicron BA.1), 38 samples from 5 different lineages from 21L clade (Omicron BA.2), 8 samples from 3 different lineages from 22A clade (Omicron BA.4), and 76 samples from 2 distinct lineages from clade 22B (Omicron BA.5). Additionally, three samples were classified as recombinant (XAG lineage). Metrics, lineage classification for each sample, and GISAID accession number are available in Supplementary Table S3.

The genomic diversity of Omicron has been reported in many studies. Nevertheless, few studies have demonstrated the diversity of synonymous mutation in this variant. Considering our dataset (*n* = 253), we analyzed this diversity and categorized the synonymous mutations into two groups (Figure 4B). The group of low-frequency mutations (purple circles) and high-frequency mutations (orange circles). In total, 858 mutations were found, although 30 synonymous mutations were present at high frequency in all samples evaluated. Four synonymous mutations were found in all assessed genomes. These mutations are found in the ORF1ab genes (C3037T, C10029T, and C14408T) and Spike (A23403G). The results indicate that, despite the high diversity of synonymous mutations, few were fixed across all lineages.

### 3.5. Omicron Introduction in MG State

Bayesian analyses based on molecular clocks estimated the origin of each of the four Omicron subvariants evaluated in our study (see original trees in Supplementary Files S1–S4). According to our analysis, BA.1 appeared worldwide in late July 2021 (95% HPD: 30 May 2021 to 3 September 2021) and was described circulating in various locations in Brazil by November. BA.2 appeared worldwide in early July 2021 (95% HPD: 27 May 2021 to 10 October 2021) and was circulating in the country by November. BA.4 appeared worldwide in early November 2021 (95% HPD: 15 April 2021 to 23 January 2022), and by July 2022, this subvariant was already circulating in Brazil. BA.5 emerged on 31 November 2021 (95% HPD: 5 June 2021 to 21 April 2022), and by July 2022, it was also already circulating in Brazil (Supplementary Files S1–S4).

**Figure 5:**
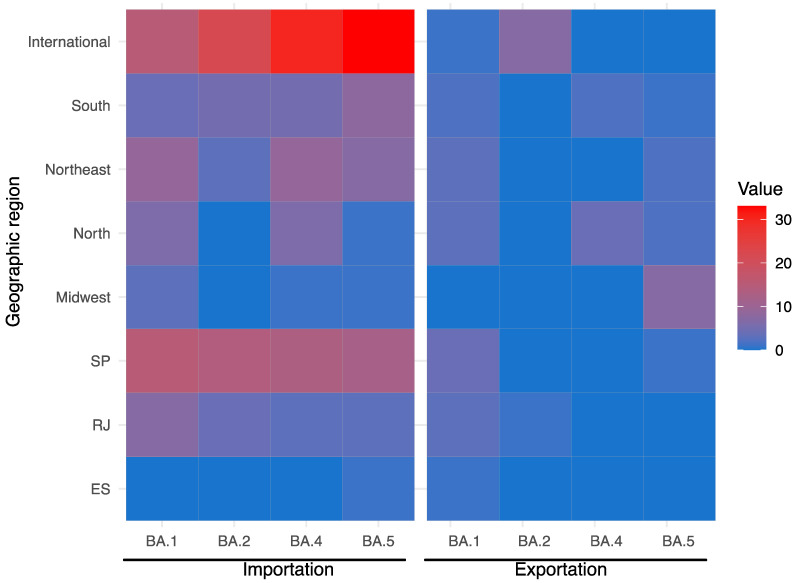
Correlation plot showing the profile of importations and exportation events for each Omicron subvariants (BA.1, BA.2, BA.4, and BA.5) in the state of Minas Gerais as evaluated in our study. Blue colors represent a lower number of events, while red colors represent a higher number of events.

Our phylodynamic analysis demonstrates that the emergence of Omicron subvariants was primarily driven by international events of importation in MG. For instance, more than 150 events were reported in our phylodynamic analysis. BA.1 introductions in MG were mainly due to international events (28 events), followed by introductions from the state of São Paulo (SP) and the northern region of Brazil (15 and 10 introductions, respectively). BA.2 introductions in MG were predominantly from international events (27 events), followed by the state of SP (12 events). BA.4 introductions in MG were also mainly from international sources (30 events), with additional introductions from SP and the northern region of the country (15 and 10 events, respectively). BA.5 was introduced into MG through multiple events, especially from international sources (35 events), and less frequently from the south and northeast regions (eight and four events, respectively). MG export events were also detected, mainly to the Brazilian midwest region (Figure 5). Our results reinforce that VOC Omicron was introduced by multiple international events, followed by interstate transmission. Furthermore, except for the BA.5 subvariant, MG did not play a significant role as an Omicron exporter to other Brazilian regions, despite its proximity to several states.

## 4. Discussion

Genomic surveillance systems have been used throughout all waves of the COVID-19 pandemic to monitor the emergence, growth, and displacement of SARS-CoV-2 variants and lineages [26]. Tracking SARS-CoV-2 variants allowed for near-real-time monitoring of circulation and changes in the number of cases and/or deaths in various regions of the world [27]. Brazil was one of the countries most affected by COVID-19; however, it also had one of the highest vaccination rates against the disease [28,29]. For instance, while the VOC Delta led to an increase in cases and deaths globally, Brazil experienced a different trend, with significant reductions in cases and deaths due to vaccination [9]. In late 2021, another VOC emerged in South Africa and drew attention from various organizations due to its high number of mutations and the subsequent rise in cases [30]. The VOC Omicron was first reported in Brazil in early November 2021 (GISAID—accessed on 19 December 2023) in the state of Rio Grande do Sul, the southern region of the country. Following its introduction, there was a significant increase in COVID-19 cases. In this study, we aimed to evaluate the spread of VOC Omicron in the state of Minas Gerais (MG), one of the most populous and interconnected states in the country (southeast region).

In MG, the rapid replacement of Delta VOCs by Omicron occurred at the end of 2021 and the beginning of 2022. The variant shift caused an increase in the number of COVID-19 cases, with EW 03 in which the Omicron VOC reached 100% of cases across the state. However, the number of deaths did not show substantial change, which may be related to the state’s vaccination profile, with nearly 90% of the population having received at least two doses of the vaccine [31].

Omicron stood out for its remarkable speed of dispersion and higher rate compared to previous variants, due to a greater number of mutations, 30 of which are in the S protein alone [32,33]. Consequently, several subvariants of Omicron emerged and were described in different parts of the globe [33,34]. To monitor the appearance of different Omicron lineages in MG, we employed a genotyping strategy to classify the samples as BA.1, BA.2, and then BA.4/BA.5 subvariants. The BA.1 was predominant for the longest period in the state. BA.2, while detected early in the year in MG, only saw a significant in cases in April (EW 14), as reported in a previous study [2]. In the middle of the year (EW 25), MG experienced another surge in cases, associated with the high frequency of two subvariants (above 80%), BA.4 and BA.5. Despite the different subvariants described in MG, the number of deaths and severe complications, such as SARS, remained low throughout the study period. This could be attributed to the vaccination status of the MG population, with the majority of cases being vaccinated with at least one booster dose (*n* = 1635—50%).

Our genotyping approach was limited in distinguishing BA.4 and BA.5 subvariants due to specific primers targeting mutations shared by both subvariants. Therefore, we performed whole-genome sequencing of a subset of positive samples from the beginning of our study, generating a total of 253 genomes. Most genomes were classified as BA.1, BA.2, BA.4, or BA.5, but three recombinant samples were reported between BA.1 and BA.2. The recombinant XAG has already been described in previous studies, circulating at low frequency in MG [2,35].

Given the extensive repertoire of mutations in Omicron, we analyzed the frequency of synonymous mutations in all genomes generated in our study. More than 250 mutations were found, but only four were fixed in the genomes evaluated. Synonymous mutations do not alter the encoded amino acid and are typically not considered for variants and lineages classification. However, recent studies have suggested that these mutations may play a critical role in the virus’s evolution, enhancing its adaptation to host codons and increasing the efficiency of protein synthesis used in viral particles during infection [36,37]. Previous studies have demonstrated that mutations in VOC Omicron may contribute to vaccine resistance, monoclonal antibodies evasion, and increased transmissibility [38,39].

COVID-19 vaccines facilitate the production of neutralizing antibodies and may also increase titers in individuals with a history of infection. As neutralization titers decrease over time and protective immunity acquired from a prior SARS-CoV-2 infection is not assured with the emergence of Omicron, concerns about vaccine effectiveness have risen. Studies have observed a decrease in neutralizing antibodies, suggesting reinfections and reduced effectiveness and treatment with monoclonal antibodies and vaccine. Nevertheless, vaccines continue to provide protection against moderate and severe cases [40,41]. In Brazil, the reinfection rate during Omicron wave was six times higher than in previous waves (compared with the variants Gamma, Zeta, and Delta). This highlights the need for continuous booster doses to enhance and prolong protection against the disease. The age group between 25 and 60 years had the highest frequency of reinfections, potentially due to the vaccination schedule and virus exposure [42].

Our phylogenetic analyses revealed the influence of the state of São Paulo (SP), and international introduction events in the establishment and spread of Omicron subvariants in MG. For BA.1 and BA.2, a previous study [2] also found SP and international events to be crucial for the establishment of these variants in MG. The increase in people movement between countries and between states through various means of transportation is a major factor facilitating the spread and establishment of distinct lineages in MG, along with the reduction in individual protection measures such as the mandatory mask usage and frequent use of 70% ethanol in public environments. In this context, genomic surveillance is a valuable tool for monitoring the lineage establishment and spread in regions with high traffic of people, goods, and services, such as the case of MG.

Our study has some limitations. It was not possible to characterize samples from all EWs in all RHUs used. Additionally, the synonymous mutations profile was based on a smaller fraction of genomes compared to those available in public databases. Despite these limitations, the integration of epidemiological and genomic surveillance enabled real-time monitoring of Omicron subvariants in MG. The results described in our study were also shared in real time with the Minas Gerais State Health Department.

## 5. Conclusions

Our study assessed the epidemiological situation based on genomic data in 15 RHUs in the state of Minas Gerais through PCR-genotyping, whole-genome sequencing, and phylodynamics analysis. A total of 3481 samples were genotyped, allowing us to monitor almost in real time the detection of the first cases of each Omicron subvariants and their dissemination throughout the state (BA.1, BA.2, BA.4, and BA.5). Epidemiological data showed that most cases had been vaccinated with at least two doses, which may be associated with a reduction in the number of deaths and the development of severe cases of the disease. We sequenced 253 new genomes, revealing the presence of other lineages at low frequency, as well as the genomic diversity of more than 250 mutations in the Omicron subvariants. Finally, the phylogenetic analysis indicated that international events were primarily responsible for the introduction and dispersion of Omicron subvariants in the state. The results of this retrospective study are essential for understanding the evolutionary dynamics of SARS-CoV-2 and for establishing public health policies and epidemiological interventions in the state.

## Figures and Tables

**Table 1:** Number of SARS-CoV-2 cases classified by sex, age, clinical outcome, and subvariant.

		**Lineages**			
**Characteristic**	**Overall. N = 3.435**	**No BA.2. N = 2.493**	**BA.2. N = 351**	**Delta. N = 591**	***p*** **-Value^2^**
** Sex**					0.152
Female	2.029 (59%)	1.491 (60%)	210 (60%)	328 (55%)	
Male	1.406 (41%)	1.002 (40%)	141 (40%)	263 (45%)	
** Death for SARS**	86 (2.5%)	45 (1.8%)	0 (0%)	41 (6.9%)	**<0.001**
** Age groups**					**0.060**
>60	774 (23%)	530 (21%)	95 (27%)	149 (25%)	
0–29	885 (26%)	657 (26%)	84 (24%)	144 (24%)	
30–60	1.776 (52%)	1.306 (52%)	172 (49%)	298 (50%)	
		**Age groups**			
**Characteristic**	**Overall. N = 3.482**	**0–29. N = 891**	**30–60. N = 1.798**	**>60. N = 793**	***p*** **-Value** ^2^
** Sex**					**<0.001**
Female	2.055 (59%)	525 (59%)	1.127 (63%)	403 (51%)	
Male	1.427 (41%)	366 (41%)	671 (37%)	390 (49%)	
Death for SARS	86 (2.5%)	1 (0.1%)	13 (0.7%)	72 (9.1%)	**<0.001**

n (%); Pearson’s Chi-squared test.

## Data Availability

All Omicron consensus genome sequences characterized in this study have been deposited on GISAID and are publicly available (Supplementary Table S3). The Supplementary Tables and phylogeny are available on our GitHub repository page: https://github.com/LBI-lab/SARS-CoV-2_phylogenies.

## Supplementary Materials

The following supporting information can be downloaded at: https://www.mdpi.com/article/10.3390/microorganisms12091745/s1, Supplementary Table S1. Dataset composition for each phylogeographic inference. The inference for each subvariant (BA.1, BA.2, BA.4, and BA.5) was made using a proportional set of sequences of each Brazilian geographic region and the genomes generated in our study. Supplementary Table S2. Genotyping and epidemiological information about the samples used in our study. Supplementary Table S3. Sample information, metrics sequencing, and GISAID accession number for the genomes generated in our study. Supplementary File S1. BA.1 phylogeographic inference. Supplementary File S2. BA.2 phylogeographic inference. Supplementary File S3. BA.4 phylogeographic inference. Supplementary File S4. BA.5 phylogeographic inference.
